# Anti-inflammatory Activities of Sulfated Polysaccharides From Ethanol Crude Extract of Spyrida Species Red Seaweed

**DOI:** 10.7759/cureus.50284

**Published:** 2023-12-10

**Authors:** Shweta Mary Reddy, Vasugi Suresh, Sivaperumal Pitchiah, Balachandran Subramanian

**Affiliations:** 1 Physiology, Saveetha Dental College and Hospitals, Saveetha Institute of Medical and Technical Sciences, Saveetha University, Chennai, IND; 2 Prosthodontics, Saveetha Dental College and Hospitals, Saveetha Institute of Medical and Technical Sciences, Saveetha University, Chennai, IND

**Keywords:** highly efficient, denaturation of bovine serum albumin, anti-inflammatory, polysaccharides, red seaweed

## Abstract

Introduction: The extracts derived from red seaweed have shown characteristics that may reduce inflammation. The abovementioned effects can potentially provide positive outcomes in managing inflammatory illnesses, including arthritis, inflammatory bowel disease, and other skin problems.

Aim: The polysaccharides were isolated from the *Spyrida* species. The water-soluble polysaccharides were extracted and fractionated from several Indian seaweeds (Red) using a simple, cost-effective approach. Anti-inflammatory effects were further evaluated and validated by FTIR and FESEM analyses.

Materials and methods: FT-IR and FESEM were used to assess the structural features of polysaccharides and the surface morphology. In addition, the red seaweed species of the genus *Spyrida*, which includes polysaccharides, was shown to significantly inhibit the denaturation of bovine serum albumin (BSA), further proving that the substance has anti-inflammatory qualities.

Results: In this work, an assay to suppress protein activity was utilized to investigate the potential anti-inflammatory effects of polysaccharides derived from *Spyrida. *As predicted, increasing concentrations of the extract, ranging from 25 to 100 µg/ml, led to a rise in the percentage of inhibited protein denaturation.

Conclusion: A statistically significant difference was found between these findings and those obtained with aspirin, a commonly used non-steroidal anti-inflammatory medicine (NSAID). The red algae that grow in the shallow waters of the southern Indian Ocean may be used in medicine.

## Introduction

In recent decades, marine algae have been recognized as distinct and appealing prospects in the disciplines of biochemistry and pharmacology compared to other marine bacteria and other species. The existence of complex and ordered sulfated polysaccharides has led to the discovery of a vast diversity of marine algae species. These polysaccharides inhibit the replication of enveloped viruses. Red algae produce derivatives such as the phycocolloid carrageenan and lectin Griffiths in, while green algae (called Ulvans) and brown algae (called fucoidans) yield sulfated polysaccharides that could potentially be used to treat SARS-CoV. Red seaweeds are interesting possibilities for dental uses due to their distinct bioactive components and characteristics. Antimicrobial bioactive substances are found in red seaweed. These chemicals can potentially impede the development of oral infections, aiding in avoiding dental caries and periodontal disorders.

Researchers have looked at the antimicrobial and antifungal effects of red seaweed extracts. Bioactive chemicals present in some red seaweed preparations aid tissue regeneration and repair. Oral operations, periodontal treatments, and oral wound healing may all benefit from these qualities. Red seaweeds like agar and carrageenan are harvested for their polysaccharides, which are then included in dental impression materials. These materials make it possible to create lifelike dental prostheses and restorations from precise impressions. Oral inflammation and gingivitis may be controlled with the help of red seaweed's anti-inflammatory qualities. Anti-inflammatory substances may reduce the redness, swelling, and pain brought on by a number of oral disorders. In Southeast Asia, seaweed has long served as a nutritional staple and an integral part of traditional substances used in developing medicines [1-3]. Several compounds found in seaweed are widely investigated, and cutting-edge pharmaceutical components in red, brown, and green seaweeds play a significant role in human diets. Several biologically active substances are found in seaweeds, including those with anti-inflammatory, antimicrobial, anti-obesity, and anti-coagulant properties. Water-soluble polysaccharides have drawn the greatest interest among these substances due to their lack of toxicity and wide range of bioactivities, including their anti-diabetic, anti-cancer, and antioxidant properties [4-8].

Polysaccharides derived from brown and red macroalgae might be employed as renewable therapeutic agents and multifunctional foodstuffs [9,10]. Carrageenan and red algae agar are the most significant polysaccharides that have found extensive use in therapeutic applications. The structural features of polysaccharides, including their molecular shape, glycosidic interactions, size, degree of sulfation, sulfate distribution pattern, and component monosaccharide ratio, vary significantly in their biological qualities, according to the previous research literature. Inflammation, which occurs when the immune system responds to potentially harmful stimuli, might be beneficial. However, escalating acute inflammation into chronic inflammation may lead to illnesses involving inflammation. According to studies by [11-15] and others, oxidative stress-mediated inflammation is crucial in the beginning and pathogenesis of many chronic illnesses. Studies have shown that naturally occurring marine compounds lower the risk of oxidative stress-induced inflammation [16]. For instance, it has been demonstrated that photoprotons and Phyto furans from the red algae Gracilaria longissimi exhibit anti-inflammatory effects on endothelial cells [17-19]. In obese rats, a dietary polysaccharide from the red algae Eucheuma cottonii reduced pro-inflammatory cytokine production and cartilage deterioration brought on by osteoarthritis. Sulfated polysaccharides from the marine alga Gracilaria Caudata inhibited inflammation in in-vivo research to avoid tissue damage.

Similarly, brown algae stopped LPS-stimulated cells' oxidative stress-induced inflammation. The data above amplifies the ability of several marine red algae species to reduce inflammation [20]. In keeping with the findings mentioned earlier, the MEC in this investigation showed a remarkable anti-inflammatory impact in lowering the denaturation of BSA.In most circumstances, inflammation results from the body's immune system responding to cellular oxidative stress. Therefore, free radical scavenging by MEC likely prevented oxidative stress, leading to its anti-inflammatory benefits.

The current research focused on the isolation and development of new water-soluble polysaccharides (WSPs) from chosen red Indian seaweeds obtained from the coastal areas of the Kanyakumari District, Tamil Nadu, India, as well as to assess their nutritional values and chemical makeup as well as medicinal uses.

## Materials and methods

Spyrida species red seaweed was gathered from the coastline of Kanyakumari District, Tamil Nadu, India. Spyrida species were cleaned carefully with running water to eliminate any contaminants adhering to the surface of the species. The seaweed was washed with distilled water and air-dried in the shade for 4-7 days. The air-dried seaweed was crushed into a fine powder and utilized for further investigation. All the other compounds used in this investigation were of analytical quality and obtained from Sigma-Aldrich, India.

Preparation

The following procedure was done to extract the polysaccharides from Spyrida species using hot water. The Spyrida red seaweed powder was heated to reflux in double-distilled water (1:5 w/v) for a duration of 2 h at a temperature of 85 ºC under reduced pressure. We used centrifugation at 100 g for 10 minutes to separate the residue from the extract. This was done after collecting the supernatant, adding three volumes of 99.5% ethanol (v/v), and cooling it to 4ºC. To dissolve the polysaccharide flake, it was taken and dissolved in distilled water. After centrifugation, we collected the polysaccharide in flakes and monitored the extraction of minerals using conductimetry. We repeated the washing and precipitating of the minerals in ethanol five times. Following a 48-hour freeze-drying process at 55ºC, the resulting product was resolubilized in ultra-pure water. To get the crude sulfated polysaccharide, previously stored samples were redissolved in distilled water, dialyzed using a membrane with a molecular weight cut-off of 6-8 kDa, and freeze-dried.

The yield was determined using the equation shown below:

Sulfated polysaccharide yield (mg/g) = sulfated polysaccharide dry weight (mg)/dry weight of seaweed material (g).

The inhibition of heat-induced protein denaturation (IDP) was studied utilizing in vitro research strategies. The data were then statistically evaluated, and a plot was created using graphical tools and the protein denaturation inhibition technique: Bovine serum albumin and the test extraction made up the test solution. Aspirin was used as the reference. The pH of each solution was adjusted to 6.3. The samples were incubated for 20 minutes at 37°C, then for 30 minutes at 57°C. 2.5 cc of phosphate buffer was poured after cooling. At 416 nm, a spectrophotometer examined the specimens (Milton Roy). The following equation was used to determine the protein denaturation inhibition percentage: Percentage of inhibition: Control OD − (Sample OD/Control OD) × 100

OD extract - optical density of extract; control- optical density of control

## Results

**Figure 1:**
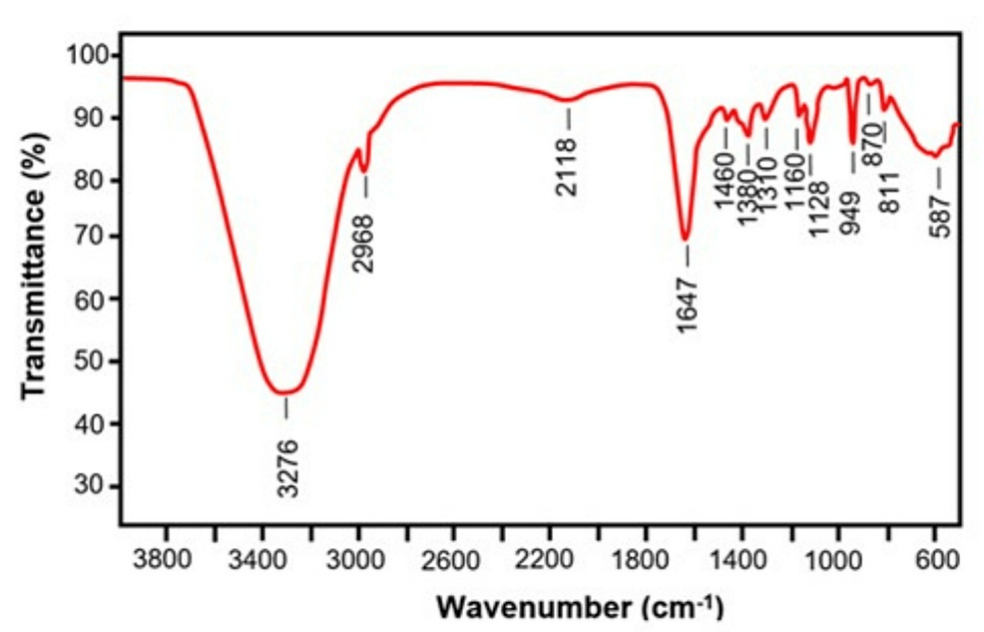
FTIR spectra of ethanolic extract of red Seaweed.

Chemical bonds have been linked to certain vibrational frequencies, and those frequencies have been used to assign peaks. The many different types of functional groups inside the polysaccharide might be more easily identified with the help of a reference book or academic journal article. Fourier transform infrared (FTIR) analysis can give information about the spectral data that may help us understand how a polysaccharide is structured. The degree of branching, the presence of glycosidic connections, and the presence of substituents are all aspects to consider. The FTIR spectra of Spyrida sp. are shown in Figure 1. There was a synthesis of red seaweed fractions. It was determined that the absorption bands at 3276 and 2968/cm [21,22] correspond to the OH-stretching and CH-asymmetric polysaccharide vibrations, respectively. Spyrida sp. has a low electrolytic potential. Absorption signals at 1460/cm and 1647/cm, attributable to (-COO-) carboxylate groups and (C=O) ester carbonyl groups in red seaweed, respectively, were shown to be authentic [21,23]. A polysaccharide similar to agar was seen in FTIR spectra [24,25], with characteristic bands at 1160, 1128, 949, and 870/cm.

**Figure 2:**
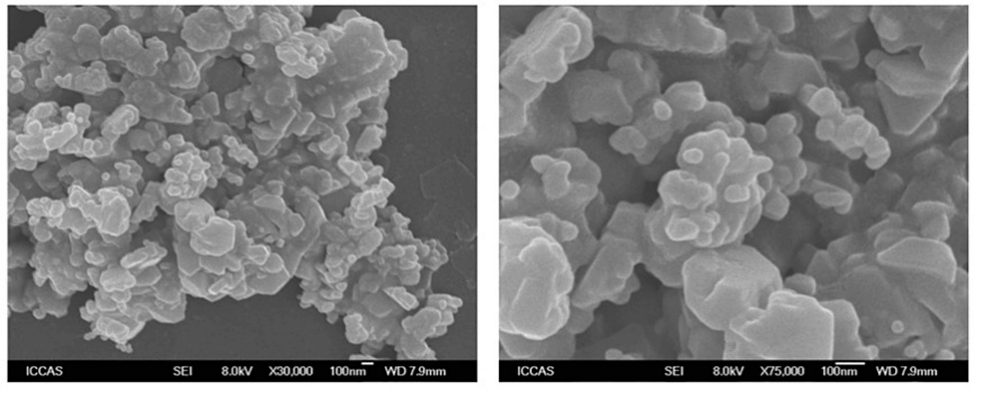
FESEM images of polysaccharide from ethanolic extract of red Seaweed at different magnifications (a) 30 K and (b) 75 K.

Field emission scanning electron microscopy (FESEM) images can be used to deduce the size and shape of polysaccharide particles. Polysaccharides may appear in the body as irregularly shaped particles. Particle size estimation is an essential feature of several disciplines, including drug delivery, food science, and materials research. Figure 2 shows the surface morphology of polysaccharides. A smooth and homogeneous surface of a polysaccharide may indicate effective purification and processing, while a rough or porous surface may indicate the presence of impurities or structural flaws. Inspecting the surface of the polysaccharide for defects such as wrinkles, pores, and cracks can assess its integrity. Polysaccharides may exhibit aggregation or clustering behaviors. Images obtained using FESEM may reveal whether polysaccharide particles gravitate toward uniform dispersion or aggregation. Many applications, such as pharmaceutical formulations and food products, place a premium on particle dispersion and stability, making a thorough understanding of aggregation behavior crucial. Figures 2a-2b show the surface morphology of polysaccharides extracted from red seaweed ethanolic extract at two different magnifications. The image depicts polysaccharides as many flakes of varying sizes, all with high porosity. The typical flake size ranges from 250 to 500 nm.

**Figure 3:**
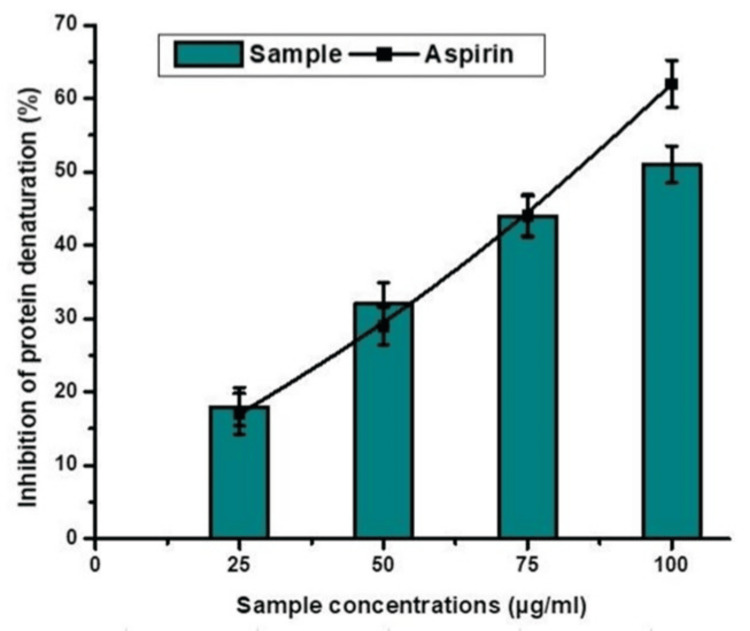
BSA Protein denaturation using polysaccharide from ethanolic extract of red Seaweed.

A polysaccharide produced from an ethanolic extract of red seaweed can render bovine serum albumin (BSA) ineffective by disrupting its native structure. To prepare a stock solution of Bovine Serum Albumin (BSA) in a buffer solution, first determine the desired concentration. Concentrations of bovine serum albumin (BSA) vary from 1 to 10 mg/mL; the experiment's aims determine the concentration utilized. Make a series of reaction solutions in separate test tubes. Each reaction mixture should include the same amount of BSA, such as 1 mL of the BSA stock solution and varying amounts of the red seaweed polysaccharide extract. To provide a thorough comparison, it is suggested that buffer solution and bovine serum albumin (BSA) be included in control tubes. Incubate the reaction mixtures at a predetermined temperature (often between 30 minutes and 1 h) and duration (typically between 37°C and room temperature). Incubation allows BSA and the polysaccharide extract to better interact with one another. The acquisition of absorbance measurements at different wavelengths is crucial for monitoring changes in the structural conformation of proteins. Denatured BSA may have altered absorbance spectra. The experimental reactions were compared to a control group where BSA without polysaccharide was used. Carefully analyze the results to determine the extent of BSA denaturation caused by the addition of the red seaweed polysaccharide extract. Statistical analysis may be required for the measurement of denaturation (Figure 3).

The anti-inflammatory effects of a polysaccharide from ethanolic red seaweed standard are presented in Table 1. The capacity for polysaccharide from ethanolic red seaweed standard to inhibit BSA from denaturing was used to evaluate its anti-inflammatory properties. As the BSA protein concentration of non-denatured BSA increased with increasing dosage of a red seaweed extract, the SEC drastically reduced the denaturation of BSA in a concentration-dependent direction, suggesting that less BSA is denatured and more non-denatured BSA remains in the reaction medium, resulting in a greater in protein concentration. Non-denatured BSA concentrations were 52.78 μg/ml of total BSA at a 100 μg/ml concentration of ethanolic red seaweed, similar to the 61.48 μg/ml of non-denatured BSA per microgram of entire BSA reported with aspirin (standard).

**Table 1:** Inhibition of protein denaturation

Concentration (μg/ml)	Percentage of inhibition of protein denaturation (%)	St. Error	Aspirin (control)%	St. Error
Control	0	0	0	0
25	18	2.6	17	2.8
50	32	2.9	29	2.6
75	44	2.7	44	2.9
100	51	2.5	62	3.2

## Discussion

Polysaccharides derived from red seaweeds, such as carrageenan and agar, have the potential to regulate the immune system's inflammatory response. Because these things can change how immune cells work, like how they make cytokines and how many immune cells they make, they may help keep inflammatory processes in check. Numerous polysaccharides derived from red seaweed have a high concentration of antioxidants, facilitating the mitigation of oxidative stress inside the human body. The presence of oxidative stress has been shown to be linked to the occurrence of inflammation.

The ability of polysaccharides to scavenge free radicals has been seen to aid in reducing inflammation. Pro-inflammatory substances like prostaglandins, leukotrienes, and inflammatory cytokines cannot be made as easily when polysaccharides from red seaweed are present. The suppression of inflammation may be facilitated by lowering the concentrations of these mediators. It has been shown that certain polysaccharides derived from red seaweed, particularly those present in Porphyra species, have the potential to promote gastrointestinal well-being. Because changes in the gut microbiota have been linked to the development of chronic inflammation, keeping the gut healthy might help lower inflammation throughout the body. Some polysaccharides from red seaweed may be able to stop the release of histamine, a biologically active molecule that plays a role in allergic and inflammatory reactions. By inhibiting the production of histamine, these substances may assist in alleviating inflammatory reactions.

Antioxidants are efficacious in safeguarding the human body against the detrimental effects induced by reactive oxygen species. Concerns about health and the possible harm of synthetic antioxidant lipids like butyl hydroxyanisole (BHA) and butylated hydroxytoluene (BHT), which are used in many treatments, have made people more interested in creating therapies that use natural antioxidants [25]. Different kinds of crude seaweed extracts, like acetonic, aqueous, methanolic, and ethanolic extracts [26], have been shown to be antioxidants in the past. These extracts have been found to contain phenolic compounds, pigments, polysaccharides, fatty acids, and peptides, which are believed to contribute to their antioxidant activities [27]. The Red algae are known to synthesize a wide range of biologically active metabolites, such as alkaloids, polyketides, cyclic peptides, polysaccharides, phlorotannins, diterpenoids, sterols, quinines, unsaturated lipids, and glycerol. According to Al-Saif et al. [28], these metabolites have a wide range of biological activities. Carotenoids, glycerol, alginates, and carrageenan are some of the metabolites used in the pharmaceutical industry to treat and control oxidative stress-related diseases in humans. The conditions above include cancer, diabetes, inflammation, allergies, and various bacterial, parasitic, and fungal diseases.

The possible antioxidant properties of seaweeds have been a subject of interest for an extended period. For instance, Yan et al. conducted a screening study to assess the DPPH-scavenging capability of 27 seaweed species originating from China. To get the most out of the whole biomass, the researchers used a method that involved extracting it using chloroform, ethyl acetate, acetone, methanol, and water [29]. Previous studies have shown the use of red seaweed's bioactive properties due to its rich assortment of phenolic compounds, amino acids, pigments, and polysaccharides, particularly in the form of a specialized fucoidan extract. Previous studies have established a correlation between consuming seaweed extracts and their potential to induce depressive and analgesic effects on the central nervous system. Additionally, these extracts have been shown to possess hypoglycemic properties and exhibit anti-inflammatory characteristics.

Inflammation is an innate immune response triggered by several stimuli, including physical damage, exposure to harmful chemicals, or microbial invasion. Enzymes and signaling transcription factors are key components of anti-inflammatory processes. The enzymes COX-1 and COX-2 have been the subject of thorough investigation in the context of inflammatory diseases, and their correlation with the occurrence of illnesses has been shown. Furthermore, the inflammatory process leads to the release of lysosomal enzymes, resulting in tissue destruction. The extracellular activity of these enzymes is believed to be associated with acute and chronic inflammation. Stabilizing the lysosomal membrane plays a crucial role in regulating the inflammatory response by effectively limiting the release of lysosomal components. The HRBC membrane has similarities to the lysosomal membrane, and the observed stability of the extract suggests that it may have a stabilizing effect on lysosomal membranes [30].

Limitation

The antioxidant activity of polysaccharides extracted from red seaweed may be influenced by the technique used for extraction. The establishment of standardized extraction processes is necessary in order to get consistent outputs. However, variances in extraction techniques might result in divergent findings. Although an increasing corpus of scholarly research exists on the antioxidant benefits of polysaccharides found in red seaweed, the availability of well-designed human clinical studies remains restricted. Further investigation is required to ascertain the efficacy and safety of these substances within a real-world context.

## Conclusions

Our comprehensive research shows that red seaweed polysaccharide is anti-inflammatory. Red seaweed Spyrida contains polysaccharides with antioxidant, antiviral, and antibacterial properties. Our work identified and characterized Spyrida polysaccharides. The current work extracted and fractionated water-soluble polysaccharides from Red Indian seaweed species using a simple, cost-effective method. FT-IR and FESEM were utilized to examine and characterize the polysaccharide structure and surface morphology. The red seaweed Spyrida species, which is high in polysaccharides, stopped bovine serum albumin (BSA) from denaturing, which suggests it might have anti-inflammatory properties. We tested Spyrida polysaccharides for their anti-inflammatory activity by inhibiting protein denaturation. Extract concentration showed a favorable correlation with protein denaturation inhibition.

The proportion of protein denaturation inhibition increased from 25 to 100 µg/ml of extract. The extract's results were compared to those of aspirin, a clinically recommended non-steroidal anti-inflammatory medicine, to assess their relevance. A statistically significant difference was seen between the groups. Red algae seen on South Indian Sea coastlines has therapeutic properties. The present research examines the anti-inflammatory activities of red seaweed polysaccharides using several experimental methods. The polysaccharide showed anti-inflammatory properties, reducing oxidative stress markers. This shows that it may reduce inflammation-related oxidative damage. The study's findings are crucial to developing natural anti-inflammatory drugs. Red seaweed polysaccharide offers considerable promise for future medical and nutraceutical research. A future study may examine the molecular processes that cause red seaweed polysaccharide's anti-inflammatory effects. Additionally, this compound's dosing regimens must be optimized. Additionally, clinical trials are needed to assess the feasibility of applying these results to humans.

## References

[1] Rajapakse N, Kim SK (2011). Nutritional and digestive health benefits of seaweed. Adv Food Nutr Res.

[2] Rodrigues Ely, Tilvi Supriya, C.G. Naik (2004). Antimicrobial activity of marine organisms collected off the coast of South East India. J Exp Mar Biol Ecol.

[3] Roshan A, Jothipriya A, Arivarasu Arivarasu (2020). Antifungal activity of tulsi and turmeric assisted copper nano particle. Plant Cell Biotechnol Mol Biol.

[4] Pereira L, Critchley AT (2020). The COVID 19 novel coronavirus pandemic 2020: seaweeds to the rescue? why does substantial, supporting research about the antiviral properties of seaweed polysaccharides seem to go unrecognized by the pharmaceutical community in these desperate times?. J Appl Phycol.

[5] Lee OH, Yoon KY, Kim KJ, You S, Lee BY (2011). Seaweed extracts as a potential tool for the attenuation of oxidative damage in obesity-related pathologies. J Phycol.

[6] Nwosu F, Morris J, Lund VA (2011). Anti-proliferative and potential anti-diabetic effects of phenolic-rich extracts from edible marine algae. Food Chem.

[7] Liu J, Hafting J, Critchley AT, Banskota AH, Prithiviraj B (2013). Components of the cultivated red seaweed Chondrus crispus enhance the immune response of Caenorhabditis elegans to Pseudomonas aeruginosa through the pmk-1, daf-2/daf-16, and skn-1 pathways. Appl Environ Microbiol.

[8] Mohamed Mohamed, S. S., Hashim Hashim, S.N. S.N., Rahman Rahman, H.A. H.A. (2012). Seaweeds: a sustainable functional food for complementary and alternative therapy. Trends Food Sci Technol.

[9] Liu X, Zhang M, Liu H, Zhou A, Cao Y, Liu X (2018). Preliminary characterization of the structure and immunostimulatory and anti-aging properties of the polysaccharide fraction of Haematococcus pluvialis. RSC Adv.

[10] Hu DJ, Cheong KL, Zhao J, Li SP (2013). Chromatography in characterization of polysaccharides from medicinal plants and fungi. J Sep Sci.

[11] Peng Y, Bishop K S, Ferguson L R (2018). Screening of cytotoxicity and anti-inflammatory properties of feijoa extracts using genetically modified cell models targeting TLR2, TLR4 and NOD2 pathways, and the implication for inflammatory bowel disease. Nutrients.

[12] Schramm A, Matusik P, Osmenda G, Guzik TJ (2012). Targeting NADPH oxidases in vascular pharmacology. Vascul Pharmacol.

[13] Rieshy V., Priya J., Arivarasu L. (2020). Enhanced antimicrobial activity of herbal formulation mediated copper nanoparticles against clinical pathogens. Plant Cell Biotechnol Mol Biol.

[14] Kim YJ, Kim EH, Hahm KB (2012). Oxidative stress in inflammation-based gastrointestinal tract diseases: challenges and opportunities. J Gastroenterol Hepatol.

[15] Hulsmans M, Van Dooren E, Holvoet P (2012). Mitochondrial reactive oxygen species and risk of atherosclerosis. Curr Atheroscler Rep.

[16] Vishaka S, Sridevi G, Selvaraj J (2022). An in vitro analysis on the antioxidant and anti-diabetic properties of Kaempferia galanga rhizome using different solvent systems. J Adv Pharm Technol Res.

[17] Martínez Sánchez S, Domínguez-Perles R, Montoro-García S (2020). Bioavailable phytoprostanes and phytofurans from Gracilaria longissima have anti-inflammatory effects in endothelial cells. Food Funct.

[18] Padmapriya A, Preetha S, Selvaraj Selvaraj (2022). Effect of Carica papaya seed extract on IL-6 and TNF-α in human lung cancer cell lines-an In vitro study. Res J Pharm Technol.

[19] da Silva FR, E Silva Conceição Pinto M, de Carvalho França LF (2019). Sulfated polysaccharides from the marine algae Gracilaria caudata prevent tissue damage caused by ligature-induced periodontitis. Int J Biol Macromol.

[20] Obuli GKS, Jothi PA, Narayanan L, Kumar R, Devi G (2020). Controlling of oral pathogens using turmeric and tulsi herbal formulations mediated copper nanoparticles. Plant Cell Biotechnol Mol Biol.

[21] Hentati F, Barkallah M, Ben Atitallah A (2019). Quality characteristics and functional and antioxidant capacities of algae-fortified fish burgers prepared from common barbel (Barbus barbus). Biomed Res Int.

[22] Ben Hlima H, Dammak M, Karkouch N (2019). Optimal cultivation towards enhanced biomass and floridean starch production by Porphyridium marinum. Int J Biol Macromol.

[23] Chiovitti A, Bacic A, Craik D (1998). A pyruvated carrageenan from Australian specimens of the red alga Sarconema filiforme. Carbohydr Res.

[24] Lajili S, Ammar HH, Mzoughi Z, Amor HB, Muller CD, Majdoub H, Bouraoui A (2019). Characterization of sulfated polysaccharide from Laurencia obtusa and its apoptotic, gastroprotective and antioxidant activities. Int J Biol Macromol.

[25] Faiez H, Cédric D, Christine G J (2020). Structural features and rheological properties of a sulfated xylogalactan-rich fraction isolated from Tunisian Red seaweed Jania adhaerens. Appl Sci.

[26] Amarowicz R, Naczk M, Shahidi F (2012). Antioxidant activity of crude tannins of Canola and Rapeseed hulls. J Am Oil Chem.

[27] Tenorio-Rodriguez PA, Murillo-Álvarez JI, Campa-Cordova ÁI, Angulo C (2017). Antioxidant screening and phenolic content of ethanol extracts of selected Baja California Peninsula macroalgae. J Food Sci Technol.

[28] Múzquiz de la Garza AR, Tapia-Salazar M, Maldonado-Muñiz M (2019). Nutraceutical potential of five Mexican brown seaweeds. Biomed Res Int.

[29] Al-Saif SS, Abdel-Raouf N, El-Wazanani HA, Aref IA (2014). Antibacterial substances from marine algae isolated from Jeddah coast of Red sea, Saudi Arabia. Saudi J Biol Sci.

[30] Z. Yi, Y. Ya, Y. Liang, B. Zeng (2008). In vitro antioxidant and antimicrobial activities of the extract of Pericarpium Citri Reticulatae of a new Citrus cultivar and its main flavonoids. LWT - Food Sci Technol.

